# Validation of the clutter image rating (CIR) scale among psychiatric outpatients in Singapore

**DOI:** 10.1186/s12888-016-1125-x

**Published:** 2016-11-17

**Authors:** Vathsala Sagayadevan, Ying Wen Lau, Clarissa Ong, Siau Pheng Lee, Siow Ann Chong, Mythily Subramaniam

**Affiliations:** 1 Research Division, Institute of Mental Health, 10 Buangkok View, Singapore, 539747 Singapore; 2 Present address: Department of Psychology, Utah State University, 2810 Old Main Hill, Logan, UT 84322-2810 USA; 3 Present address: Department of Psychology, The Chinese University of Hong Kong, Shatin, NT, Hong Kong SAR, People’s Republic of China

**Keywords:** Clutter, Hoarding, Validation, SI-R

## Abstract

**Background:**

The Clutter Image Rating (CIR) Scale though extensively used to assess hoarding behavior, has mainly been validated in Western populations.

**Methods:**

The current study sought to validate the CIR in a sample of psychiatric outpatients (*n* = 500) in Singapore. Convergent and divergent validity as well as inter-observer reliabilities between participant CIR and interviewer-rated CIR were calculated.

**Results:**

The CIR performed fairly in identifying participants with and without hoarding problems according to the Savings Inventory Revised (SI-R). The CIR composite demonstrated good convergent validity with the SI-R clutter subscale, the SI-R total and the Activities of Daily Living Scale for Hoarding (ADL-H) scale and discriminant validity with the Beck Anxiety Inventory (BAI), Beck Depression Inventory (BDI), and Quality of Life Enjoyment and Satisfaction Questionnaire – Short Form (Q-LES-Q-SF) scale.

**Conclusions:**

Findings add valuable knowledge to the utility of the CIR in an Asian population.

## Background

Hoarding is characterized by difficulty discarding possessions due to strong urges to save items, accumulation of clutter which precludes the use of living spaces for their intended purposes, excessive acquisition behaviors, and the presence of significant distress or impairment in functioning [1–3]. The accurate and timely assessment of hoarding symptoms is crucial given that excessive acquisition behaviors often result in substantial clutter which consequently lead to significant social and occupational impairments (e.g., loss of job, family dysfunction) and in severe cases, fire hazards or even death [2, 4, 5].

Prevalence of hoarding behavior has been approximated to range between 2 and 6% [3, 6, 7]. Mueller et al. [7], for instance, found approximately 4.6% of the German general population to have compulsive hoarding (previously considered a diagnostic feature of obsessive-compulsive disorder (OCD)) whereas Samuels et al. [3] estimated the prevalence of compulsive hoarding to be nearly 4% in the community. Studies have shown that hoarding symptoms are co-morbid with several disorders, in particular, anxiety [8–10] and depressive disorders [11–13]. Hamblin et al. [14] found children with elevated hoarding symptoms to score significantly higher on measures of anxiety and to have higher rates of major depressive disorder (MDD) than those without hoarding. Likewise, Frost et al. [15] found OCD hoarding patients to score higher on anxiety and depression compared to non-hoarding OCD and anxiety disorder patients.

To date, several instruments have been developed to assess the severity of hoarding symptoms and associated functional impairment [9, 16]. These include self-report questionnaires such as the Saving Inventory - Revised (SI-R) [17] which examines the three main domains of hoarding behavior: difficulty discarding, excessive clutter, and excessive acquisition, the Activities of Daily Living in Hoarding Scale (ADL-H) [18] which assesses the degree to which clutter interferes with one's daily activities, and the Clutter scale (CS) which enlists a series of questions to examine the extent of clutter in each target room (i.e., kitchen, living room, bedroom) [9]. Others include semi-structured interviews such as the Hoarding Rating Scale-Interview (HRS-I) [19] which assesses the three main features of hoarding (clutter, difficulty discarding, and excessive acquisition) as well as the distress and interference caused by the hoarding behavior.

Despite the well-established psychometric properties of these hoarding measures, the main methodological issue posed by these instruments exists in the variability in the perception of what constitutes “clutter” which potentially limits the accuracy of self-reports of clutter [9]. To resolve the problem associated with self-reports of the clutter dimension, Frost et al. [9] developed a visual analogue scale-Clutter Image Rating (CIR) Scale which aimed to measure the severity of clutter in compulsive hoarding. For instance, in the original validation study by Frost et al. [9], self-report measures assessing clutter (e.g., SI-R) were found to differ significantly depending on the location in which they were completed, with those completed in the clinic having significantly higher scores than those completed at home. This difference however, was not observed for the CIR, suggesting that it may be less likely to be affected by an over-reporting bias [9, 19]. The composite CIR also showed a strong inter-observer reliability [9] with a reported participant/experimenter correlation of 0.78; according to Cohen [20], an absolute value of r of more than 0.5 is classified as strong correlation.

Although the CIR has been extensively used in studies to assess hoarding behavior, its utility has mainly been established in Western populations [9, 21]; to our knowledge, no such validation studies have been conducted in Asia. Possible cultural differences in hoarding symptoms have been found in studies which have validated the SI-R scale [22]. Yorulmaz and Dermihan [22] for instance, found the mean score of total hoarding symptoms on the SI-R in a Turkish community sample to be lower than that of Western subjects. In comparing US and Chinese student samples, Timpano et al. [23] found the Chinese sample to endorse greater hoarding symptoms compared to the US sample on the SI-R. Furthermore, the Chinese sample was found to primarily endorse saving beliefs centered on usefulness and wastefulness in relation to hoarding as compared to their Western counterparts who endorsed a wider range of beliefs. This difference was attributed to cultural differences in manifestation of hoarding tendencies whereby the Chinese may feel more strongly about beliefs on saving compared to their Western counterparts [23].

Similarly, in validating the factor structure of the SIR in a psychiatric outpatient population in Singapore, Lee et al. [24] found the SI-R scale, though consistent with Frost et al.’s [17] three factor structure: clutter, difficulty discarding, and acquisition, to be empirically under-identified. The removal of two items on self-control from the 23-item scale in turn, resulted in a marginal improvement in model fit; a finding consistent with Timpano et al. [23]. One possible reason cited, was the possibility of differential emphasis placed on the role of self-control in collectivistic cultures [25], particularly in Chinese culture whereby discarding of possessions may be viewed as a wasteful habit rather than a maladaptive behavior resulting from attachment to possessions [24].

According to the cognitive behavioral model of hoarding [23, 26], three basic deficits: information processing problems, beliefs related to emotional attachments to possessions, and the reinforcing impact of emotions and avoidance are posited to play a critical role in hoarding. In accordance with this, cultural differences in beliefs associated with hoarding (i.e., wastefulness, usefulness) are likely to have an impact on hoarding behavior and the level of clutter endorsed by individuals.

Singapore is multicultural country in South-East Asia with a population of 5.5 million; of its residents, 74.3% are of Chinese descent, 13.3% are Malay, 9.1% are of Indian descent, and 3.3% belong to other ethnic groups [27]. Given the cultural differences in hoarding tendencies and symptoms observed in past validation studies of the SIR, it is unclear if the CIR, developed in a Western sample will be valid in multicultural populations [23].

Furthermore, the unique housing structure in Singapore warrants validation of the scale in the local context given that the majority of the residents reside with family members in public housing developed by the Housing Development Board (HDB) [28]. These apartments are located within close proximity of one another whereby individuals often have shared common areas; this is likely to have a moderating effect on the hoarding behavior and clutter level of individuals.

In view of this, we sought to validate the CIR in a sample of psychiatric outpatients in Singapore to determine its utility in the local population. Specifically, we aimed to: (1) evaluate the accuracy of the CIR in identifying hoarding status as classified by the SIR, and (2) establish the convergent and divergent validity of the CIR with related and non-related constructs.

## Method

### Sample

The Clutter Image Rating (CIR) scale was validated based on the data collected from a study assessing hoarding behaviour among psychiatric outpatients. Convenience sampling was used to recruit five hundred outpatients seeking treatment at the Institute of Mental Health (IMH), a tertiary hospital in Singapore specializing in psychiatric care between May 2014 and April 2015. The study was approved by the institutional ethics committee (National Healthcare Group, Domain Specific Review Board). To be eligible for the study, participants had to be: (1) at least 21 years old, (2) have a primary DSM-IV diagnosis of any anxiety disorder (AD; including OCD), any depressive disorder (DD), schizophrenia (SZ), or pathological gambling (PG), (3) able to comprehend and complete the questionnaires in English, and (4) cognitively capable of providing informed consent. Participants with co-morbid conditions were also included in the present study. Patients with cognitive deficits were excluded from the study [29]. The majority of the sample was male (56.4%) and of Chinese ethnicity (70.2%). Ten percent of the sample was of Malay ethnicity, 13.4% were Indian, and 6.4% belonged to other ethnicities; the mean age of participants was 35.3 years (range = 21–69).

### Procedure

All participants signed an informed consent form prior to completion of the study questionnaire packet, which comprised a demographic questionnaire and the self-report measures: Clutter Image Rating (CIR) Scale, Saving Inventory - Revised (SI-R), Saving Cognitions Inventory (SCI), Beck Anxiety Inventory (BAI), Beck Depression Inventory-II (BDI-II), Activities of Daily Living Scale for Hoarding (ADL-H), and Quality of Life Enjoyment and Satisfaction Questionnaire – Short Form (Q-LES-Q-SF). The administration of questionnaires was not part of their routine care and was performed solely for the purpose of research. Participants completed the questionnaires while waiting for their appointment or after their appointment. Upon completion of the study questionnaires, participants were reimbursed an inconvenience fee of 30 SGD. In addition, two trained research assistants visited houses of participants who were agreeable to a home visit (n = 46; as indicated on the consent form) at a convenient time within a few weeks of completing the questionnaire and separately rated the rooms (i.e., kitchen, living room, bedroom) based on the CI-R. Research assistants were blinded to the diagnosis of the patients (i.e., they were not aware if the patients had significant hoarding symptoms or not). Participants were compensated with 10 SGD following the completion of the home visit.

Further details of the study can be found in previous articles by Ong et al. [29] and Lee et al. [24].

### Instruments

#### Clutter image rating (CIR) scale

The CIR consists of three pages of nine color photos representing a range of clutter in the living room (LR), bedroom (BR) and kitchen (K). Patients were asked to select the picture “that most accurately reflects the amount of clutter in your room”. Scores ranged from 1 (least cluttered) to 9 (most cluttered). A mean composite score ranging from 1 to 9 was calculated across the three rooms for each individual. Both composite and room-by-room scores were examined in this study. A cut-off score of 4 or higher was used to indicate significant clutter requiring clinical attention [9]. Previous validation studies on the CIR have demonstrated good psychometric properties [9]. Internal consistency for the current sample was good (α = .84).

#### Saving inventory - revised (SI-R)

The *Saving Inventory-Revised* (SI-R) [17] is a 23-item self-report measure comprising three subscales: difficulty discarding, clutter, and excessive acquisition. Items were scored between 0 (None) and 4 (Almost all/Complete), with higher scores indicating greater hoarding severity. Internal consistency, test-retest reliability, and convergent and divergent validity have been established [10, 17]. A SI-R cut-off score of 41 or higher indicates clinically significant hoarding symptoms [30]. This cut-off score was selected on the basis that it maximized sensitivity (=0.959) and specificity (=0.931) for the scale (Unpublished data, cited in Tolin et al. [30]). Internal reliabilities for the full scale and three subscales were good to excellent in the current sample (Cronbach’s α values ranged from .80 to .93).

#### Saving cognitions inventory

The *Saving Cognitions Inventory* (SCI) [26] is a 24-item self-report measure that examines maladaptive beliefs about and emotional attachment to possessions. It consists of four subscales: emotional attachment, control, responsibility, and memory. Participants are asked to indicate the extent to which they had each thought when deciding whether or not to dispose something in the past week. An example of an item is “I could not tolerate it if I were to get rid of this”. Scores ranged from 1 (not at all) to 7 (very much). The full scale and four subscales showed satisfactory internal consistency in the present study (α values ranged from .78 to .96).

#### Beck anxiety inventory

The *Beck Anxiety Inventory* (BAI) [31] is a 21-item self-report measure that evaluates distress associated with common symptoms of anxiety. Items are rated from 0 (not bothered at all) to 3 (severely bothered). Internal consistency for the current sample was excellent (α = .95).

#### Beck depression inventory

The *Beck Depression Inventory-II* (BDI-II) [32] is a 21-item self-report measure that assesses depressive symptoms. Items are rated from 0 to 3, with higher scores indicating greater severity. Internal consistency for the current sample was excellent (α = .94).

#### Activities of daily living scale for hoarding

The *Activities of Daily Living Scale for Hoarding* (ADL-H) [18] is a 15-item self-report measure that assesses the degree of functional impairment in daily living activities due to clutter. Responses are scored from 1 (can do it easily) to 5 (unable to do), and are averaged across items, with higher scores indicating greater impairment. Internal consistency for the current sample was excellent (α = .96).

#### Quality of life enjoyment and satisfaction questionnaire – short form

The *Quality of Life Enjoyment and Satisfaction Questionnaire – Short Form* (Q-LES-Q-SF) [33] is a 16-item scale that assesses enjoyment and satisfaction across various life domains, including mood, social relationships, and economic status. Higher scores indicate higher self-rated quality of life. Internal consistency for the current sample was excellent (α = .93).

## Results

### Sample description

Participants were classified into one of the diagnostic groups on the basis of their primary diagnosis: depressive disorders (DD; *n* = 153; 30.6%), schizophrenia (SZ; *n* = 150; 30.0%), anxiety disorders (AD; *n* = 144; 28.8%), and pathological gambling (PG; *n* = 53; 10.6%). Within the AD group, 9.8% had OCD (*n* = 49), 7.6% had anxiety disorder not otherwise specified (NOS) (*n* = 38), 5.0% had panic disorder (*n* = 25), 4.0% had generalized anxiety disorder (GAD; *n* = 20), 2.2% had social phobia (*n* = 11), and 0.2% had specific phobia (*n* = 1). In the DD group, 24.8% had major depressive disorder (*n* = 124), 3.4% had dysthymia (*n* = 17), and 2.4% had depressive disorder NOS (*n* = 12). Forty seven (9.4%) had a comorbid depressive disorder, 33 participants (6.6%) had a comorbid anxiety disorder, and 9 (1.8%) had comorbid anxiety and depressive disorders.

### Classification of hoarding status

As the presence of clinically significant hoarding problems was not a prerequisite for our study, the SI-R scores were used as a reference to understand the hoarding characteristics in our sample. The SI-R has previously been validated in a predominantly Chinese sample and was deemed as an appropriate comparison tool for the CIR [23, 24]. It was previously established that a cutoff score of 41 or higher on the SI-R would indicate clinically significant hoarding symptoms [30]. Applying this to our sample, 147 participants (30.2%) were divided into the hoarding group and the remaining 340 into the non-hoarding group. Thirteen participants were excluded as they did not fully complete the SI-R questionnaire, hence their SI-R scores were not calculated and they were not classified into either of the two groups. Descriptive information for each of the study measures is provided in Table 1.

**Table 1:** Descriptive information for study measures^a^

		Mean	SD	Minimum-Maximum	Mean inter-item correlation
SI-R total		30.8	15.97	0-77	0.37
SCI total		69.17	31.61	24-168	0.48
ADL-H		1.40	0.63	1-5	0.62
BAI		16.94	13.35	0-61	0.46
BDI-II		19.64	13.77	0-62	0.43
Q-LES-Q-SF		54.48	18.06	0-100	0.50
CIR Composite		1.65	0.85	1-9	0.65
	Living Room	1.60	1.02	1-9	
	Bedroom	1.67	1.01	1-9	
	Kitchen	1.70	0.93	1-9	

^a^ *n* ranges from 434 to 487

A Receiving Operating Characteristic (ROC) curve was constructed to evaluate the accuracy of the participant-rated CIR in identifying hoarding status as classified by the SI-R. The accuracy is measured by the Area under the ROC curve (AUC); an area of 1 represents that the CIR is perfect and an area of 0.5 represents that the CIR is poor in terms of identification. The reported AUC was 0.753, implying a relatively fair performance of the CIR in identifying participants with and without hoarding problems according to the SI-R. Compared to 30.2% of participants who were identified as having clinically significant hoarding symptoms on the SI-R, only 2.9% of participants were classified as having significant hoarding symptoms based on the CIR. The results were similar for each of the three CIR rooms (living room = 0.701, bedroom = 0.713, kitchen = 0.694).

The inter-correlations among the three CIR rooms were high (Bedroom-Living Room *r* = 0.66, Bedroom-Kitchen *r* = 0.61, Living Room-Kitchen *r* = 0.65) and the CIR had good internal consistency (α = 0.84).

### Convergent and discriminant validity

The CIR and ADL-H scores were non-normally distributed; hence, Spearman’s rho correlation coefficients were used to determine the strength of associations among the variables.

The CIR composite was significantly correlated with the SI-R clutter subscale (*r* = 0.56), the SI-R total (*r* = 0.54) and the ADL-H scale (*r* = 0.51) (Table 2), which demonstrated convergent validity of the CIR on the clutter construct. In contrast, the correlations were weaker with measures of related constructs such as SCI (*r* = 0.42) and other subscales of the SI-R (r from 0.27 to 0.47) (Table 2). The CIR composite score was also weakly correlated with non-related constructs - BAI (*r* = 0.253), BDI (*r* = 0.229) and Q-LES-Q-SF (*r* = -0.239) (Table 3). In this regard, the CIR also demonstrated good discriminant validity.

**Table 2:** Correlations among CIR Composite, SI-R, ADL-H and SIR

	CIR composite	CIR Living Room	CIR Bedroom	CIR Kitchen	SI-R total	SI-R clutter	SI-R difficulty discarding	SI-R acquisition	SCI total	SCI control	SCI responsibility	SCI emotional attachment	SCI memory
CIR Living Room	0.844												
CIR Bedroom	0.842	0.646											
CIR Kitchen	0.852	0.616	0.589										
SI-R total	0.543	0.485	0.473	0.441									
SI-R clutter	0.564	0.503	0.469	0.456	0.904								
SI-R difficulty discarding	0.467	0.400	0.424	0.388	0.888	0.713							
SI-R acquisition	0.390	0.371	0.334	0.305	0.853	0.626	0.698						
SCI total	0.417	0.364	0.384	0.322	0.626	0.520	0.607	0.567					
SCI control	0.267	0.213	0.213	0.227	0.418	0.325	0.404	0.401	0.799				
SCI responsibility	0.362	0.344	0.317	0.282	0.619	0.519	0.585	0.578	0.926	0.697			
SCI emotional attachment	0.398	0.342	0.366	0.308	0.610	0.507	0.595	0.554	0.949	0.684	0.824		
SCI memory	0.412	0.351	0.356	0.324	0.601	0.521	0.571	0.530	0.896	0.615	0.815	0.812	
ADL-H	0.513	0.440	0.441	0.432	0.566	0.582	0.488	0.414	0.379	0.285	0.344	0.383	0.374

All correlations had *p*-values <0.001

**Table 3:** Correlations of CIR Composite with BAI, BDI and Q-LES-Q-SF

	CIR composite	CIR living room	CIR bedroom	CIR kitchen	BAI	BDI-II
CIR Living Room	0.844					
CIR Bedroom	0.842	0.646				
CIR Kitchen	0.852	0.616	0.589			
BAI	0.253	0.199	0.245	0.199		
BDI-II	0.229	0.181	0.212	0.181	0.703	
Q-LES-Q-SF	-0.239	-0.228	-0.227	-0.184	-0.588	-0.667

All correlations had *p*-values <0.001

### Inter-rater reliabilities

To establish reliability of the CIR, inter-observer reliabilities were calculated for participant CIR and interviewer-rated CIR on the subset of 46 participants.

Cohen’s k was run to first determine consistency between the two interviewers on their rating of hoarding status using the CIR. There was fair to moderate agreement between the two interviewers for each of the three rooms (Kitchen: k = 0.482; *p*-value <0.001; 95% CI [0.272, 0.691]), (Bedroom: k = 0.372; *p*-value = 0.001; 95% CI [0.162, 0.582]), (Living room: k = 0.507; *p*-value <0.001; 95% CI [0.309, 0.705]), as well as the composite CIR (k = 0.382; *p*-value <0.001; 95% CI [0.225, 0.539]). Consistencies between non-corresponding rooms were significant with a lower level of agreement.

Inter-rater reliability between interviewers and participants were calculated using Spearman’s rho correlation coefficient to understand how closely the participants’ CIR ratings in the clinic matched the interviewers' CIR ratings in the house. The average of the interviewers’ ratings was used for the calculation. It was noted that the ratings were left skewed, hence, the coefficients were corrected for restriction of range using Thordike Case II [34] to obtain a more accurate relationship. The corresponding rooms and composite scores correlated significantly except for the correlation between participant CIR-Bedroom and interviewer CIR Kitchen (*r* = .351, *p* > .05) and the correlation between participant CIR-Bedroom and interviewer CIR-living room (*r* = .193, *p* > .05) (Table 4).

**Table 4:** Correlations of CIR ratings by participants with CIR by interviewers

	Interviewer CIR			
Participant CIR	Kitchen	Bedroom	Living Room	Composite
Kitchen	0.611^b^	0.524^a^	0.504^a^	0.604^b^
Bedroom	0.351	0.445^a^	0.193	0.441^a^
Living Room	0.503^b^	0.336^a^	0.414^b^	0.482^b^
Composite	0.515^b^	0.464^b^	0.367^a^	0.541^b^

^a^Correlation is significant at the 0.05 level (2-tailed) *n* = 44

^b^Correlation is significant at the 0.01 level (2-tailed)

## Discussion

The current study sought to validate the Clutter Image Rating (CIR) Scale among psychiatric outpatients in Singapore. In seeking to establish the accuracy of the CIR in identifying hoarding status (i.e., individuals with/without clinically significant hoarding symptoms) as classified by the SI-R, the CIR revealed a relatively fair performance. Compared to 30.2% of participants who were identified as having clinically significant hoarding symptoms on the SI-R, only 2.9% of participants were classified as having significant hoarding symptoms based on the CIR. The SI-R mean score in the current study closely mirrored those reported by Timpano et al. [23] in their Chinese sample, whereby more hoarding symptoms were endorsed by the Chinese sample on the SI-R compared to their western counterparts. It is possible that potential cultural differences (e.g., greater importance accorded to saving beliefs) could have resulted in the endorsement of more hoarding symptoms on the SI-R which may have inflated the score on this scale.

Likewise, the discrepancy in identification could be due to the two scales measuring different aspects of hoarding; CIR being a measure of the consequence of hoarding (i.e., Clutter) and the SI-R being a measure of hoarding behaviors.

The instrument showed good internal consistency with high correlations among the three CIR rooms (kitchen, living room, bedroom). The scale also demonstrated good convergent validity with related measures of clutter such as the SI-R clutter subscale and the ADL-H scale. The CIR composite score was significantly correlated with all three subscales of the SI-R, particularly the clutter subscale. This was in line with Frost et al. [9] who found participants' CIR scores in the clinic and at home to be more strongly correlated with their ratings of the SI-R clutter subscale than with the other SI-R subscales. Likewise, the current results were also supported by Dozier and Ayers's [21] study among adults aged 40–87 years old. The CIR score was significantly correlated with the SI-R total and the SI-R Clutter subscale in both the late life sample (adults aged 60–87 years) and in the mid-life sample (adults aged 40–59 years) [21]. Furthermore, the CIR score was not found to be significantly related to the ‘difficulty discarding’ and ‘acquisition’ subscales of the SI-R in both their samples [21]. Dozier and Ayers [21] also noted significant associations between the CIR and the ADL-H in both their late-life and the mid-life sample, a finding that paralleled our result. This association between ADL impairment and hoarding was reflected in Ayers et al.'s [35] study whereby hoarding severity measures (including CIR) in a late life sample was found to be positively associated with ADL impairment. Hoarding participants were found to report “moderate to great difficulty” in finding important things, moving around inside the house, eating at the table, using the kitchen sink, preparing food, and sleeping in bed whereas control participants indicated moderate to great difficulty only in terms of ‘finding important items’ [35].

Conversely, the CIR also indicated good discriminant validity through weaker correlations with non-clutter related subscales and scales such as the BAI, BDI, Q-LES-Q-SF, SCI and the SI-R subscales of ‘difficulty discarding’ and ‘acquisition’. Past literature with regard to the association between hoarding and affect has generally yielded inconsistent findings [9]. As with the current study, Frost et al. [9] study, found the CIR composite score to be significantly but moderately correlated with the BAI scores. Likewise, Coles et al. [10] found a strong association between hoarding and anxiety sensitivity in a non-clinical sample of undergraduate psychology students whereby heightened levels of anxiety sensitivity was found to contribute to avoidance of discarding. In contrast to our study however, Frost et al. [9] did not find any significant associations between the CIR and BDI scores. Interestingly, our results were partially reflective of Frost et al's [9] findings in that although significant, the correlations between CIR scores and BAI were generally higher than that between CIR scores and BDI.

These findings are in line with Frost and Hartl’s [1] cognitive-behavioral model of compulsive hoarding whereby individuals may avoid discarding objects so as not to experience an unpleasant emotional state which may negatively reinforce maladaptive hoarding behaviors. This has implications in terms of treatment whereby focus can be placed on coping with negative emotional symptoms (i.e., anxiety and depressive symptoms) to better manage hoarding behaviors.

Inter-observer reliabilities between participant CIR and interviewer-rated CIR revealed significant correlations for corresponding rooms and composite scores. This was generally lower than figures reported by studies such as Frost et al. [9] which found the correlation between the CIR completed by the participant and the therapist to be high (*r* = .78) whereas, in Dozier and Ayer's [21] late life sample, the association between the participant and clinician was moderate (*r* = .54). While it is possible that the participant-rated CIR in the clinic is a good representation of their homes, the low correlation might be partly attributed to the time lag between the completion of the CIR in the clinic and the home visit which would have allowed ample time for participants to clean up their clutter. Also, a large majority of the participants in our sample were living in HDB flats (87.7% of the patients) with 69% staying with their immediate family. It is possible that clutter could be regularly monitored and disposed of by family members which prevented the buildup of clutter which could in turn have resulted in the discrepancy and low correlations between participant and interviewer ratings.

The current findings however, should be considered in view of the study limitations. There is a potential selection bias given that home visits were conducted only for a subsample of participants; it is thus possible that those who did not consent to the home visit may have had more severe hoarding behaviors. It is also important to note that both Frost et al. [9] and Dozier and Ayers [21] validated the scale among individuals who had significant hoarding problems which were rated as being 'definitely disturbing/disabling' or were diagnosed with hoarding disorder (HD) whereas our study consisted of an outpatient psychiatric population whereby the presence of significant hoarding behaviors was not a prerequisite for study participation; this could have led to some of the variability observed in results between the studies. Nevertheless, validating the CIR among an outpatient psychiatric population provides valuable insight. Based on our sample, the CIR reflected 2.9% of participants with hoarding behavior which was aligned to previous findings by Subramaniam et al. [36] who found the weighted prevalence of lifetime hoarding behavior in the Singapore population to be 2%. Although findings may not be directly comparable given that the present study was conducted in a psychiatric outpatient sample and the aforementioned in a community sample, current findings suggest that hoarding may not be more prevalent in a psychiatric sample as measured by the CIR than in a community sample. Further verification with regards to this is however required.

## Conclusions

In line with past studies, findings from the current study generally supported the utility of the CIR as being a useful measure in assessing clutter by circumventing the shortcomings associated with self-report measures which are prone to an over-reporting bias [9, 21]. Although figures yielded from the current study were lower than that reported in past validation studies, findings add valuable knowledge to the utility of CIR given the dearth of studies which have validated the CIR, particularly in an Asian population. While there is some evidence suggestive of cultural differences in terms of hoarding tendencies and endorsement of hoarding symptoms, more in-depth exploration is required in relation to this. In conclusion, given that hoarding behaviors not only endanger the safety and health of an individual but also those around them [2], our results lend support to the use of the CIR as a quick and useful measure to evaluate clutter and its associated functional impairment.

## Abbreviations

AD: Anxiety disorders
ADL-H: Activities of daily living in hoarding
AUC: Area under the ROC curve
BAI: Beck anxiety inventory
BDI: Beck depression inventory
BR: Bedroom
CIR: Clutter image rating
CS: Clutter scale
DD: Depressive disorders
DSM-IV: Diagnostic and statistical manual of mental disorders-IV
GAD: Generalized anxiety disorder
HD: Hoarding disorder
HDB: Housing development board
HRS-I: Hoarding rating scale-interview
IMH: Institute of mental health
K: Kitchen
LR: Living room
MDD: Major depressive disorder
NOS: Not otherwise specified
OCD: Obsessive-compulsive disorder
PG: Pathological gambling
Q-LES-Q-SF: Quality of life enjoyment and satisfaction questionnaire – short form
ROC: Receiving operating characteristic
SCI: Saving cognitions inventory
SGD: Singapore dollars
SI-R: Saving inventory revised
SZ: Schizophrenia
